# Treatment of Natural Rubber Skim Latex Using Ultrafiltration Process with PVDF-TiO_2_ Mixed-Matrix Membranes

**DOI:** 10.3390/polym17121598

**Published:** 2025-06-08

**Authors:** Rianyza Gayatri, Erna Yuliwati, Tuty Emilia Agustina, Nor Afifah Khalil, Md Sohrab Hossain, Wirach Taweepreda, Muzafar Zulkifli, Ahmad Naim Ahmad Yahaya

**Affiliations:** 1 Department of Chemical Engineering, Faculty of Engineering, Universitas Sriwijaya, Jalan Palembang-Prabumulih KM 32, Indralaya 30662, Indonesia; rianyzagayatri@ft.unsri.ac.id (R.G.); tuty_agustina@unsri.ac.id (T.E.A.); 2 Malaysian Institute of Chemical and Bioengineering Technology, Universiti Kuala Lumpur, Alor Gajah 78000, Malaysia; nafifah.khalil@s.unikl.edu.my; 3 Chemical Engineering Study Program, Faculty of Engineering, Universitas Muhammadiyah Palembang, Jalan A. Yani 13 Ulu Kota, Palembang 30263, Indonesia; deeyuliwati@gmail.com; 4 HICoE-Centre of Biofuel and Biochemical Research, Department of Applied Sciences, Institute of Sustainable Energy and Resources, Universiti Teknologi PETRONAS, Seri Iskandar 32610, Malaysia; sohrab.hossain@utp.edu.my; 5 Polymer Science Program, Division of Physical Science, Faculty of Science, Prince of Songkla University, Hat-Yai 90110, Thailand; wirach.t@psu.ac.th; 6 Green Chemistry and Sustainability Cluster, Branch Campus Malaysian Institute of Chemical and BioEngineering Technology, Universiti Kuala Lumpur, Alor Gajah 78000, Malaysia; muzafar@unikl.edu.my

**Keywords:** mixed-matrix membranes, PVDF, TiO_2_, PVP, natural rubber, skim latex, skim serum, concentrate latex, ultrafiltration

## Abstract

Natural rubber skim latex is commonly discarded as waste or turned into skim natural rubber products such as skim crepe and skim blocks. It is challenging to retrieve all residual rubbers in skim latex since it has a very low rubber content and many non-rubber components like protein. Manufacturers conventionally utilize concentrated sulfuric acid as a coagulant. This method generates many effluents and hazardous pollutants that negatively impact the environment. This work presents an innovative method for enhancing the skim latex’s value by employing an ultrafiltration membrane. This study aims to establish a hydrophilic PVDF-TiO_2_ mixed-matrix membrane. The skim latex was processed through a membrane-based ultrafiltration process, which yielded two products: skim latex concentrate and skim serum. Skim latex deposits that cause fouling on the membrane surface can be identified by SEM-EDX and FTIR analysis. The PVDF–PVP-TiO_2_ mixed-matrix membrane generated the maximum skim serum flux of 12.72 L/m^2^h in contrast to the PVDF pure membranes, which showed a lower flux of 8.14 L/m^2^h. CHNS analysis shows that a greater amount of nitrogen, which is indicative of the protein composition, was successfully extracted by the membrane separation process. These particles may adhere to the membrane surface during filtration, obstructing or decreasing the number of fluid flow channels. The deposition reduces the effective size of membrane pores, leading to a decline in flux rate. The hydrophilic PVDF-TiO_2_ mixed-matrix membrane developed in this study shows strong potential for application in the latex industry, specifically for treating natural rubber skim latex, a challenging by-product known for its high fouling potential. This innovative ultrafiltration approach offers a promising method to enhance the value of skim latex by enabling more efficient separation and recovery.

## 1. Introduction

Natural rubber latex (NRL) is derived from the Para rubber tree (*Hevea brasiliensis*) [1]. Natural rubber latex is a raw material used in various medical and pharmaceutical products, such as medical gloves and condoms [2,3]. Centrifugation is the primary method used by natural rubber latex manufacturers to generate concentrated latex composed of high rubber particles and by-products of skim latex in the serum phase [4]. The centrifugation of fresh latex yields skim latex as a by-product and concentrated latex as the main product [5]. Skim latex is frequently discarded as waste or processed into skim natural rubber goods such as skim blocks. The conventional procedures for processing the skim latex include centrifugation, coagulation using acids and microorganisms, incubation, and treatment with enzymes and surfactants. These processes only produce low-price skim latex products, have detrimental environmental effects, and eventually cause environmental and water pollution [6,7].

The processing of raw natural rubber latex might be revolutionized by the membrane separation method since it provides better control over the treatment rate and product quality [8]. Latex processing can efficiently transform value-added raw materials to achieve discharge status by recovering skim latex and turning it into skim latex concentrate and skim serum [2,9]. The low-value by-product known as skim latex is frequently discarded as effluent waste [10]. The rubber content from raw natural rubber skim latex is expected to be raised by using the ultrafiltration (UF) membrane separation method. Compared to the acid coagulation procedure, it is a cleaner processing alternative for raw skim latex and might produce two new raw materials: skim latex concentrate and skim serum [2]. Skim latex concentrate is considered a novel raw material with additional value. The by-product of centrifuging field latex into latex concentrates is natural rubber skim latex (NRSL) [11]. When skim latex is processed and coagulated with subpar sulfuric acid, 4–5% of the dry rubber is recovered [2]. Skim latex is processed using an environmentally friendly ultrafiltration membrane separation technique to create skim latex concentrate [12].

Recent research has demonstrated that polyvinilidene fluoride (PVDF) is one of the best materials among other polymer materials for fabricating membranes [13,14]. PVDF is a polymer film and a versatile material for various advanced applications [15]. PVDF is susceptible to fouling and is highly responsive to harsh operating conditions, including pH, pressure, and temperature. PVDF also has excellent mechanical properties, chemical resistance, and thermal stability [16]. The natural hydrophobic nature of PVDF restricts its usage; however, mixed-matrix membranes (MMMs) provide a method to overcome this limitation [17,18]. The extensive use of membrane technology necessitates the creation of membranes that are resistant to fouling. Nanoparticle (NP) integration into a polymeric membrane matrix is also known as the production of MMMs [19]. Mixed-matrix membranes outperform polymer and ceramic membranes in terms of hydrophilicity, mechanical and chemical properties, and membrane performance [13,20]. Nanomaterials and polymers are the materials used to create polymer nanocomposites, which utilize the benefits of both components. Nanomaterials with additional surface modifications have emerged as efficient sorbents due to their high surface area and strong chemical activity [21]. These materials have been exploited to develop very effective materials for environmental applications, piquing the interest of academics and businesses worldwide [22,23]. Furthermore, advancements in polymer nanocomposite materials’ properties have opened up various industrial uses. Researchers have enhanced membrane matrixes by embedding nanoparticles (NPs), forming nanoporous structures that expand the surface area [24]. Polymer nanocomposites are essential in membrane materials because they improve membrane transport characteristics and provide outstanding stability under different operating circumstances [25]. As a result, nanocomposite membranes are a new type of membrane created at the nanoscale using organic and inorganic polymeric components. They are thought to perform better than conventional membranes [23,26]. TiO_2_ is the most advantageous choice for a nanomaterial due to its notable enhancements in membrane performance and fouling reduction [27]. TiO_2_ is extensively employed as an inorganic nanoparticle in wastewater treatment due to its exceptional performance in membrane production [28]. This includes its capacity to measure contact angles, exhibit great chemical stability, and possess antibacterial properties [29]. TiO_2_ also has gained considerable attention as a high-performance material, owing to its unique attributes such as high photo-reactivity, superhydrophilicity, non-toxicity, and long-term stability [30]. TiO_2_ in membranes aids in the formation of a more open pore structure, allowing water molecules to move through more easily [31]. While previous studies have used PVDF-based membranes for filtration applications, very few studies have explored their use specifically for natural rubber skim latex, a challenging by-product with high fouling potential. Our study is among the first to report the use of PVDF-PVP-TiO_2_ mixed-matrix membranes in this specific objective for the latex industry. This study investigates how membranes from polymer nanocomposites (PVDF-TiO_2_ membranes) are used in environmental applications for skim natural rubber latex treatment.

This study proposes a novel way to increase the value of skim latex using an ultrafiltration membrane. The goal of the current study is to produce an innovative PVDF-TiO_2_-PVP mixed-matrix membrane for skim latex filtration. Using a membrane separation technique, skim latex is processed without acidification to generate skim latex product while reducing pollution from sulfuric acid, which works as a skim latex coagulant. This study expects to generate rubber products of the highest quality due to the membrane separation process by creating skim serum (permeate) and concentrated skim latex (retentate) that will increase the commercial value of skim latex compared to skim crepe or block made by coagulation (low-priced products). This membrane technology could support the use of skim latex, potentially converting a low-value industrial by-product into usable skim serum and concentrated skim latex, aligning with circular economy principles.

## 2. Materials and Methods

### 2.1. Materials

PVDF Kynar pellets were supplied by Arkema Inc. in Philadelphia, PA, USA. Merck, a company based in Rahway, NJ, USA, supplied N, N-dimethylacetamide (DMAc) in the form of a solvent. The DMAc offered had a purity level of over 99%. Evonik GmbH, located in Essen, Germany, supplied TiO_2_ nanoparticles (Degussa P_25_) with an approximate average particle size of 21 nm. Sigma-Aldrich in St. Louis, MI, USA, supplied polyvinylpyrrolidone (PVP). Natural rubber skim latex was supplied by Tavorn Rubber Industry, Co., Ltd., Hatyai, Thailand. The PVDF concentration was 16% wt, PVP at 1% wt, and TiO_2_ at 2% wt [32].

### 2.2. Skim Latex Preparation

The field latex will go through a centrifugation process, producing two components: latex concentrate and skim latex [33]. The skim latex is provided by Tavorn Rubber Industry (1982) Co., Ltd., Hatyai, Thailand. The skim latex was produced by the centrifugation process of fresh latex. And the waste skim serum from industry is conventionally produced by using the acid coagulation process with H_2_SO_4_ as the coagulant.

### 2.3. Preparation of PVDF-TiO_2_ Mixed-Matrix Membranes

Membranes were prepared using the non-solvent phase inversion method (NIPS), as depicted in our previous study [32]. A portion of the PVDF pellets was subjected to pre-drying for 24 h at a temperature of 50 °C. The solution was created by dissolving polyvinylpyrrolidone (PVP) in the dimethylacetamide (DMAc) solvent and then distributing the necessary amount of TiO_2_ into the solution. The PVDF with a mass of 16 g was dissolved in the solution, which was subsequently agitated at a speed of 450 rpm for 24 h and then heated to a temperature of 60 °C. The membrane solution was left undisturbed for 24 h before being cast, and then the membrane was submerged in water for a whole day after being cast. The membrane was allowed to dry at room temperature for 24 h. The membrane’s performance was evaluated by measuring the permeate flux following filtration.

### 2.4. Skim Latex Filtration Using Membrane Ultrafiltration Process

The performance of the membrane is commonly assessed based on its selectivity and flux rate. As Abdullah et al. [20] described, flux is the liquid flow rate per unit area through a membrane over a certain period. The flux was measured using an experimental setup of ultrafiltration equipment. Separation efficiency was assessed by conducting experiments using a flat-sheet membrane in an ultrafiltration system, with a working pressure of 1.0 bar, to quantify the permeability of the fluid across the membrane. The filtration area of the flat-sheet membrane is 0.43 cm^2^.

The membrane’s performance was evaluated by measuring the skim latex flux. The solution that flowed through was collected every 15 min for one hour to measure the permeating flux. To determine the rejection percentage, a portion of the solution was poured into a vial, and its concentration was measured using a spectrophotometer. The flux (*J*) was estimated by applying Equation (1) as reported by Ong et al. [34]:

(1) $$J=\frac{Q}{A\times t}$$

*J* is the skim latex flux (L/m^2^h), *Q* is the permeate volume in litres, *t* is the time in hours needed to achieve volume *Q*, and *A* stands for the effective membrane area in square metres. The feed volume for filtration was 1.5 L of skim latex, which recirculates in the ultrafiltration system. The TMP was fixed at 1 bar following earlier investigations. Permeate volumes were measured every 15 min during the two hours of each trial. After the trial ultrafiltration runs, the concentrated solutions were put back into the feed tank. It was necessary to send the feed back to the feed tank so that the feed amount remained constant.

### 2.5. Analysis of Skim Latex Filtration Products

The filtration of skim latex using mixed-matrix membranes will produce two materials (skim serum and skim latex concentrate). Skim serum (permeates) is characterized by flux measurement, pH, FTIR, and CHNS (carbon, hydrogen, nitrogen, and sulphur) analysis. Skim latex samples are further processed for FTIR analysis to analyze the functional group. FTIR analysis conducted with Thermo Fisher Scientific FTIR Equipment (Waltham, MA, USA). The concentrated skim latex (retentate) characteristics are analyzed using % total solid content (TSC), alkalinity, viscosity, pH, and FTIR. The procedures for determining TSC are based on (ISO 124: 2014 [35]), and for alkalinity, they are based on ISO 125: 2011 [36]. The measurement of viscosity for skim latex is based on ISO 1652: 2011 [37].

Membranes used after the skim latex filtration are characterized by FESEM-EDX to examine membrane morphology and elemental analysis. Membranes are also analyzed by FTIR to indicate alterations in the functional groups and elemental composition of polymers and other components.

## 3. Results and Discussion

### 3.1. Skim Latex Properties Analysis

The skim latex samples were obtained from Tavorn Rubber Industry Co., Ltd. (Songkhla, Thailand). The skim latex data are displayed in Table 1. Skim latex has a total solid content (TSC) of around 6.99%, a pH of 9.59, and an alkalinity of 0.244%.

A Particle Size Analyzer determined that skim latex has particles that are 459 nm in size on average. It demonstrates that the latex skimmed particle size value is still within the specified latex skim size range. This is in line with the findings of Chan et al. [11], which showed that latex skim contained small rubber particles (size 200 nm). Alex et al. [38] also mentioned that skim latex has particles with diameters between 70 and 995 nm.

### 3.2. Skim Latex After Ultrafiltration Process Using Mixed-Matrix Membrane

An ultrafiltration membrane separation technology can be employed to transform unprocessed skim NR latex into concentrated skim latex. Skim latex was efficiently concentrated by membrane separation, leading to the manufacture of concentrated skim latex. The procedure was eco-friendly and did not involve the use of harmful chemicals. Currently, advancements in membrane separation have focused on generating concentrated latex and refining process parameters rather than examining the characteristics of the resulting concentrated skim latex. This work aims to investigate the characteristics of skim serum for potential uses and to determine if concentrated skim latex is suitable for natural rubber latex applications to effectively utilize waste from natural rubber production. Thus, this study would like to analyze the characteristics of skim serum and skim latex concentrated prepared by the membrane separation procedure. The present research compared the characteristics of membrane-concentrated skim latex with raw skim latex.

#### 3.2.1. Physicochemical Properties of Skim Serum and Concentrate Skim Latex

Table 2 shows the physicochemical properties of fresh skim latex and concentrated skim latex. The concentrated skim latex has been obtained from the membrane ultrafiltration process. The total solid content of skim natural rubber latex was only 6.99%, and it slightly increases to 7.67% for concentrated skim latex after the membrane ultrafiltration process. The raw skim latex has a low solid content due to being a by-product of the centrifugation process.

The skim latex showed a lower TSC than the concentrated skim latex. The pH of fresh skim latex is 9.59. The pH does not change significantly for concentrated latex (9.34) and skim serum or permeate (9.32). The results align with previous studies, indicating that skim latex typically includes approximately 5–8% wt of rubber content [39] or roughly 3–10% wt of rubber [9], with pH around 9–10 [40,41] and alkalinity around 0.2% [42].

#### 3.2.2. Total Solid Content (TSC) of Skim Latex and Concentrated Latex

Raw skim latex generally consists of around 4–10% rubber and 90–96% other substances, such as water, proteins, and phospholipids. In the skim latex concentrate, there are many components, and one of those is water. Water is an undesired compound in the latex concentrate to determine the TSC and DRC. This theory is easy to implement because the latex concentrate just needs to be dried for two hours at 100 °C for the determination of TSC. Based on the other theory, which is that the TSC should be higher than the DRC, it can be shown that the data for all samples followed the theory that the TSC is higher than the DRC, so the data and results for sample one are acceptable and achieve the objective of the experiment. The TSC of skim latex before filtration is around 6–7%, while after going to membrane filtration, the TSC became higher, approximately 8%, while the dry rubber content (DRC) of skim latex is around 4%.

#### 3.2.3. Alkalinity and pH of Skim Latex and Concentrated Latex

The alkalinity of fresh feed (skim latex) is around 0.244%, and the alkalinity of concentrated skim latex, as a result of skim latex filtration using a mixed-matrix membrane, is around 0.4–0.55%. The alkalinity of latex measures how much of the alkaline substances, which are ammonia or KOH, are used as preservative agents in the latex concentrate for stabilization. These are also used to determine the type of latex that was used. Theoretically, low-ammonia latex can have lower alkalinity than high-ammonia latex. This is because a low volume of acid can be used to neutralize the alkali. The level of ammonia residue in skim latex is frequently higher than found in concentrated latex [43].

The alkalinity level of latex affects colour, shelf life, and colloidal stability, among other features, making different types of latex suited for different processes. An acid-based titration, as outlined in ISO 125 and other standards, is used to determine alkalinity in the latex company [44]. The pH of the new skim latex is 9.59. The pH does not change significantly for concentrated skim latex (9.34) and skim serum or permeate (9.32). The pH range of 9 to 10 suggests that there were no significant changes in pH between fresh skim latex and concentrated skim latex following the membrane separation procedure.

#### 3.2.4. Viscosity of Skim Latex and Concentrated Latex

The viscosity of the latex rose from 50 cPs for skim latex to 133.33 cPs for concentrated skim latex. The TSC had an impact on the viscosity. As the temperature of the system increases, the viscosity also rises. The TSC of fresh skim latex was slightly lower than that of concentrated latex, and it had a lower viscosity. The concentrated skim latex should have higher viscosity. This can be attributable to the increased concentrations of non-rubber and metal ions present in the concentrated skim latex. The metal ion concentration in the highly concentrated skim latex, particularly Al^3+^, Zn^2+^, Cu^2+^, Fe^2+^, and Ca^2+^, was significantly greater compared to fresh natural rubber latex [45]. The presence of metal ions could engage in reactions with the non-rubber components, resulting in the formation of crosslinking or complexes. This process ultimately leads to the thickening of the latex. Previous research has demonstrated that divalent metal ions facilitate the creation of branching sites, leading to the production of a gel and improving the storage hardening of rubber [46,47]. Metal ions could be the cause of the concentrated latex’s higher viscosity compared to skim latex, even though they have identical total solid content (TSC).

The concentration of NR latex in a field rose with the duration of storage [45]. The findings point to hydrogen bonding and chemical cross-linking as the main causes of gel formation in concentrated NR latex. These cross-links also have a crucial function in the progressive thickening that happens during extended storage. The stability of skim latex was found to be positively correlated with the duration of storage of field latex during the coagulation process. Skim latex’s phase separation was found to be proportional to the ammonia concentration and storage time [43].

### 3.3. CHNS Analysis

Nitrogen is mostly found in crude latex protein in raw natural rubber. Protein is the primary source of nitrogen in raw natural rubber, and its concentration is considered a significant quality indicator because of its importance to the raw rubber’s processing efficiency. The anti-ageing and vulcanization properties of its breakdown products are intimately tied to the longevity of raw rubber goods. Currently, measuring the nitrogen level in raw natural rubber is crucial for both product development and quality management of raw materials [48,49].

**Figure 1:**
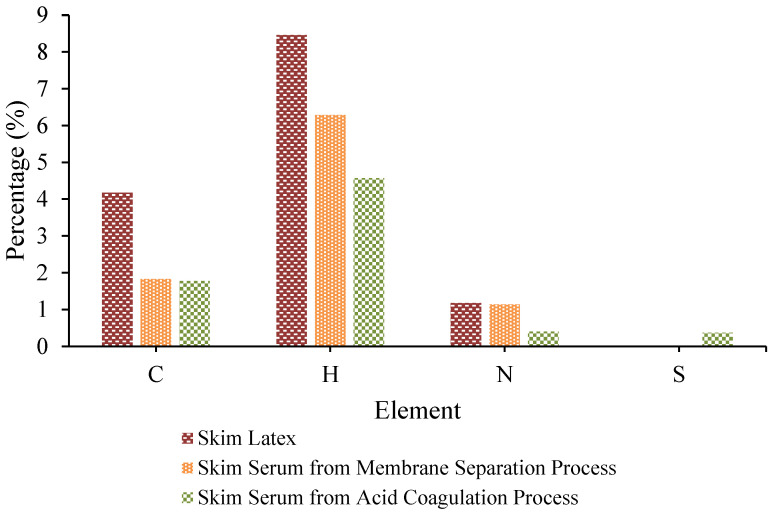
CHNS analysis of skim latex and skim serum from membrane filtration process.

In this research, the components of C, H, N, and S were analyzed using CHNS Analysis. All the details about the quantitative results of carbon, hydrogen, nitrogen, and sulphur in skim latex and skim serum are presented in Figure 1. In skim latex, the percentage of nitrogen, carbon, and hydrogen is 1.173%, 4.175%, and 8.46%, respectively. However, in skim serum obtained from the membrane filtration process, the percentage of nitrogen, carbon, and hydrogen is 1.139%, 1.828%, and 6.286%, respectively. All the percentages of the components (C, H, and N) in skim serum are lower than in skim latex. Nevertheless, the skim serum obtained by the conventional method with the acid coagulation process using sulfuric acid has also been analyzed.

The CHNS results show that carbon (1.774%), hydrogen (4.572%), and nitrogen (0.401%) elements were lower than in skim serum, resulting from the membrane separation process. It also shows that the membrane separation process successfully separated the nitrogen that represents the protein content in skim latex in a higher percentage compared to the skim serum prepared from the conventional coagulation method. Additionally, the skim serum from the membrane filtration process did not detect the sulphur element, while the skim serum from the acid coagulation process also detected the sulphur (S) component (0.367%). This is due to the utilization of a significant amount of sulfuric acid, and this is not good for the environment because it can aggravate the wastewater problem and degrade the quality of the skim.

The skim serum typically consists of approximately 4% total solids, which include nitrogen in the form of protein (0.09%), ammonium sulphate (0.26%), and potassium (0.2%) [50]. Several investigations have shown evidence of the capacity of skim latex serum to facilitate the synthesis of several beneficial compounds. The presence of vital elements, namely Mg, Fe, Mn, N, P, K, and Zn, in the serum results from the coagulation of skim latex [2,51]. These elements play a crucial role in supporting plant growth.

### 3.4. FTIR Analysis of Skim Latex and Skim Latex Concentrate

**Figure 2:**
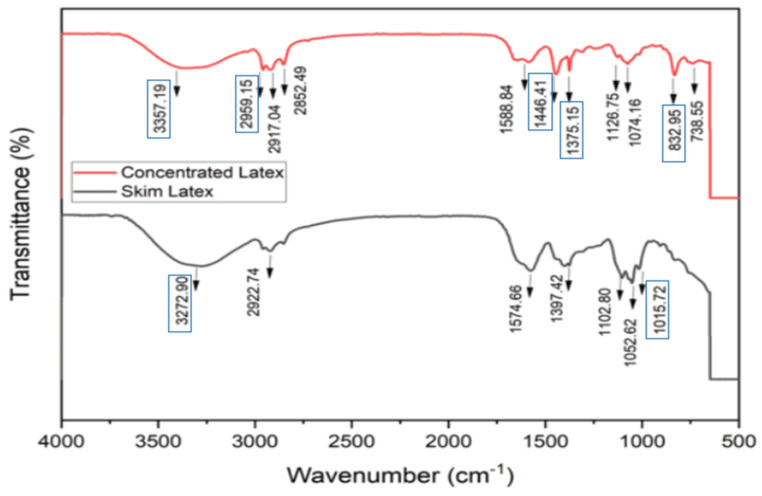
FTIR of fresh skim latex and concentrated latex.

The spectra of skim latex and concentrated latex are shown in Figure 2. The skim latex and concentrated skim latex samples show peaks at 3272 cm^−1^ and 3357 cm^−1^, respectively. Near the standard latex peaks, a strong wide band ranging from 3200 to 3500 cm^−1^ suggests the existence of hydroxyl groups. The main peaks representing the functional groups of isoprene in natural rubber are detected at the following wavenumbers: 3033, 2961, 2927, 2855, 1664, 1450, 1375, and 837 cm^−1^. Apart from isoprene, other peaks indicate the presence of oxygenated compounds, such as an amide peak at 3280 cm^−1^, a tiny amide peak at 1548 cm^−1^, and a series of peaks between 1130 and 1010 cm^−1^ [4]. The peak at around 1080 cm^−1^ is attributed to the C–O group [52]. The wave values 1647, 1540, 1447, and 1357 cm^−1^ correspond to the peaks associated with the presence of foulant amino acids and amine from rubber protein [53].

### 3.5. FTIR of Concentrated Skim Latex and Skim Serum from Membrane Filtration Process

**Figure 3:**
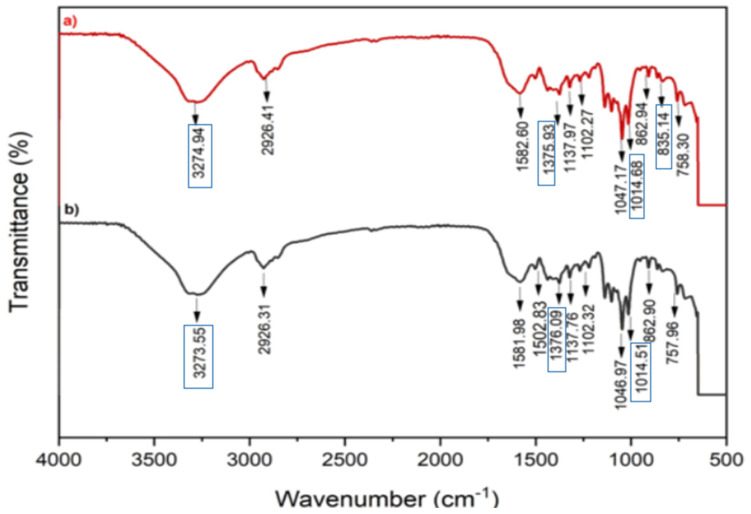
FTIR of (**a**) concentrated skim latex; (**b**) skim serum.

The spectra of skim serum and concentrated skim latex are shown in Figure 3. Skim serum FTIR spectra show the peaks at 3273.55, 2926.39, 1581.98, 1502.82, 1376.09, 1322.16, 1268.19, 1220.66, 1137.76, 1102.32, 1045.97, 1014.51, 908.76, 862.90, 757.96, and 654.55 cm^−1^. However, concentrated skim latex has principal peaks at 3275.29, 2925.77, 1581.96, 1375.79, 1321.85, 1136.82, 1101.65, 1047.52, 1014.78, 908.17, and 757.46 cm^−1^.

The C-H stretching of the CH_3_ peak was presented at 2926, while the peak at 1375 demonstrated the C-H bending of CH_3_. The peak at 1014 is associated with -O-O- in natural rubber protein alignment. Previous studies on skim serum have reported the presence of hydroxyl groups, as indicated by a strong wide band between 3200 and 3500 cm^−1^. Two peaks, one at around 1080 cm^−1^ and one at 3280 cm^−1^, were determined to represent C-O group amides [54]. The main peaks representing isoprene functional groups in natural rubber are detected at the following wavenumbers: 3033, 2961, 1664, 1450, 1375, and 837 cm^−1^. Additional peaks unrelated to isoprene are observed, showing a range of peaks between 1130 and 1010 cm^−1^ that suggest the presence of oxygenated chemicals [52]. The peaks observed at the wavenumbers of 1510–1580 cm^−1^ are indicative of the amine vibrations [55].

### 3.6. Measurement of Skim Serum Flux from Skim Latex Ultrafiltration

Skim latex serum is a residual substance derived from the processing of latex effluent in rubber manufacturing facilities, which contains a mixture of ammonia, sulphate, organic compounds, and inorganic materials [56,57]. In this study, skim latex was obtained via the ultrafiltration process using a membrane, and the filtration can produce two products: skim serum and skim latex concentrate.

**Figure 4:**
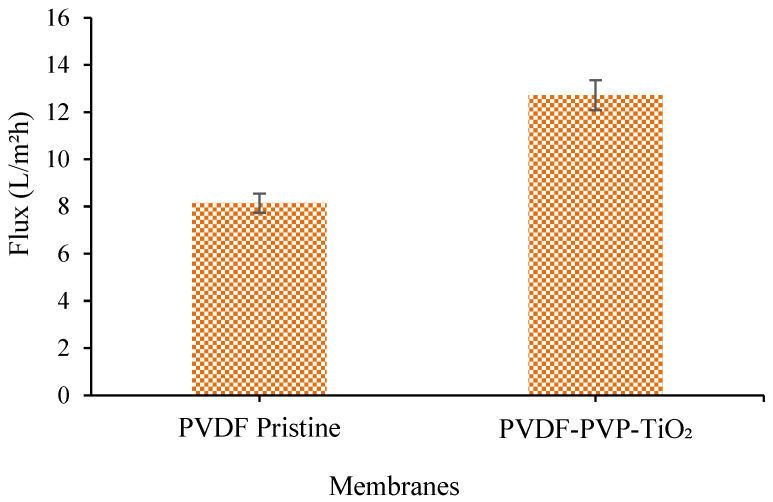
Skim serum flux measurement using PVDF pristine and PVDF-PVP-TiO_2_ membranes.

As presented in Figure 4, in comparison to the PVDF pristine membranes, the PVDF–PVP-TiO_2_ mixed-matrix membrane produced the highest skim serum flux (12.72 L/m^2^h) while the PVDF pristine membrane exhibited a lower flux of 8.14 L/m^2^h. The skim latex ultrafiltration process took two hours, with 1 bar trans membrane pressure, and the permeate was collected every 15 min for one hour.

The permeating fluxes showed that adding TiO_2_ and pore-forming agents increased the permeability and hydrophilicity of the mixed-matrix membranes [32]. The water permeate flux performance through the membrane showed that this is due to increased hydroxyl groups on the TiO_2_ surface, which improves permeate water flux and increases porosity in the membrane structure despite its more hydrophobic properties [58]. PVP membranes have higher water flux due to an increase in the thickness of the sponge-like structure on the support membrane, which drastically alters the resistance to water transport [59].

The choice of membrane material and its characteristics can also influence flux. Pristine PVDF membranes may have limited resistance to fouling and particle deposition compared to modified membranes, such as PVDF–TiO_2_ with PVP. The hydrophilicity, anti-fouling properties, and surface roughness can be increased by adding TiO_2_ nanoparticles and PVP to the PVDF membrane, leading to higher flux rates by reducing fouling and particle deposition [22]. The low flow reported in skim latex filtering utilizing membranes can be ascribed to multiple causes, such as the deposition of skim latex particles and fouling [60,61]. Skim latex adsorption and deposition on the surface of the membrane might lead to pore blockage in the membranes, which can account for the gradual decrease in flow. The deposition of skim latex particles happens because skim latex contains suspended particles, including proteins, lipids, and other impurities. During filtration, these particles can deposit onto the membrane surface, blocking or reducing the available pathways for fluid flow. This deposition causes a decrease in the effective size of the membrane pores, resulting in a drop in the flux rate.

### 3.7. Membrane Characterization After Filtration of Skim Latex

#### 3.7.1. FESEM-EDX of Membrane After Skim Latex Filtration

FESEM images and EDX analysis can be utilized to accurately detect and characterize fouling or deposits that are associated with fouling on the membrane surface. The composition of fouling layers may differ based on the characteristics of the filtrate and the conditions under which the filtration process is conducted. The presence of contaminants or deposits on the membrane surface can be observed, depending on the filtration technique employed and the characteristics of the substance being filtered. These can be identified and analyzed using EDX.

FESEM is a viable technique for visualizing the surface morphology of a PVDF-TiO_2_-PVP membrane after the process of skim latex filtration. This may provide data regarding the dimensions and spatial arrangement of the latex particles existing on the membrane’s surface. The provided data can be utilized to enhance the filtering procedure’s efficiency and prolong the membrane’s durability.

**Figure 5:**
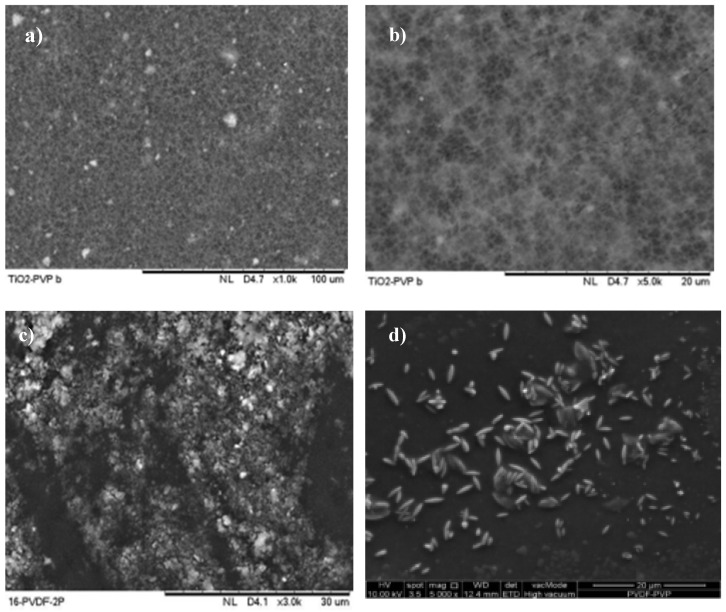
FESEM of PVDF- PVP-TiO_2_ flat-sheet membrane: (**a**) before skim latex filtration with 1 k magnifications; (**b**) before skim latex filtration with 5 k magnifications; (**c**) After skim latex filtration with 3 k magnifications; (**d**) after skim latex filtration with 5 k magnifications.

This study used a PVDF-PVP-TiO_2_ mixed-matrix membrane with a 16% wt PVDF concentration. As presented in Figure 5a,b, before filtration, the created membrane exhibited a smooth surface devoid of any deposition of materials. Pores with interconnected structures were identified on the surface of all membranes at a magnification of 1.00 and 5.00 k×. According to Teow et al. [62], it can be inferred that the selection of TiO_2_ nanoparticles does not substantially influence the alteration of membrane shape. The membrane was modified by including TiO_2_ nanoparticles, leading to the observable arrangement of TiO_2_ on the membrane’s surface. The appearance of these nanoparticles can vary, either as small, uniformly distributed particles or as clusters, contingent upon the synthesis technique employed and how TiO_2_ is distributed within the membrane.

In contrast to the membrane post-filtration, the surface exhibits evident fouling or deposition of skim latex, as presented in Figure 5c,d. The membrane with 16 wt.% of PVDF showed that the agglomerated skim latex developed on the membrane’s surface. After latex filtration, it becomes evident that latex particles or aggregates have adhered to the membrane surface. The dimensions, morphology, and spatial arrangement of these latex particles can offer valuable information about the efficacy of the filtration procedure. When employing a PVDF-PVP-TiO_2_ membrane for the filtration of skim latex, it is observed that the latex particles have the propensity to be entrapped on the membrane’s surface. This phenomenon may reduce the membrane’s permeability and elevation in the pressure differential across the membrane.

The membrane surface showed significant particle deposition due to a subsequent reduction in surface negativity. This reduction further enhanced the likelihood of particles contributing to coagulation attachment and cake formation [63]. Teow et al. [62] concluded that the decrease in flow is affected by both the molecular shape and size, and the roughness of the membrane. According to their research, smoother ultrafiltration membranes are less likely to foul than microfiltration membranes with rougher surfaces. Hence, it has been suggested that tactics centred on altering the surface characteristics of membranes could be utilized to reduce fouling [64,65]. In general, membranes that exhibit hydrophilic characteristics provide enhanced resistance to fouling and increased flux recovery.

Numerous studies have extensively documented the advantageous impacts of membranes based on nanoparticles in reducing fouling, including self-cleaning and anti-fouling capabilities [66,67]. The main factor can be attributed to the enhancement of the membrane’s hydrophilicity through the incorporation of nanoparticles. The membrane functionality associated with NPs is contingent upon the ability of NPs to disperse throughout the membrane matrix [62]. The gradual build-up of material on the membrane surface ultimately caused a size reduction in the pores, resulting in increased physical retention and, subsequently, higher hydraulic resistance.

The PVDF membrane can be made less fouling-prone by adding hydrophilic TiO_2_ nanoparticles to its matrix. The higher attraction between TiO_2_ and water leads to increased hydrophilicity. Studies have demonstrated that the ability of TiO_2_ nanoparticles to prevent fouling is a result of the water shielding effect generated by the hydrophilic –OH groups on the surface of the membrane [68]. Filtration procedures frequently experience membrane fouling, which results from particles, impurities, or chemicals in the feed solution building up on the membrane’s surface or inside its pores. The findings suggest that TiO_2_ addition enhances hydrophilicity. Nonetheless, pore-clogging and high surface roughness characteristics may offset this improvement in hydrophilicity, potentially leading to diminished anti-fouling qualities.

#### 3.7.2. Energy Dispersive X-Ray (EDX) of Membranes After Filtration

Any changes in surface features or composition may impact its effectiveness. Skim latex, typically derived from natural rubber, primarily contains elements such as hydrogen (H), carbon (C), and oxygen (O). The precise elemental composition may differ based on the source of the latex and the presence of any additional substances or contaminants. Composting waste from the concentrated rubber latex business shows promise as a sustainable and eco-friendly approach [69]. The predominant element in the latex film was carbon, at 94.46% by weight and 99.16% by atom. The primary constituent of the polymer is cis-1,4-polyisoprene units, which consist mainly of carbon components. The presence of elemental zinc was identified using EDX analysis, which was attributed to the incorporation of zinc oxide (ZnO), zinc diethyldithiocarbamate (ZDEC), and zinc 2-mercaptobenzothiazole (ZMBT) during the manufacturing process of the natural rubber film [70].

**Figure 6:**
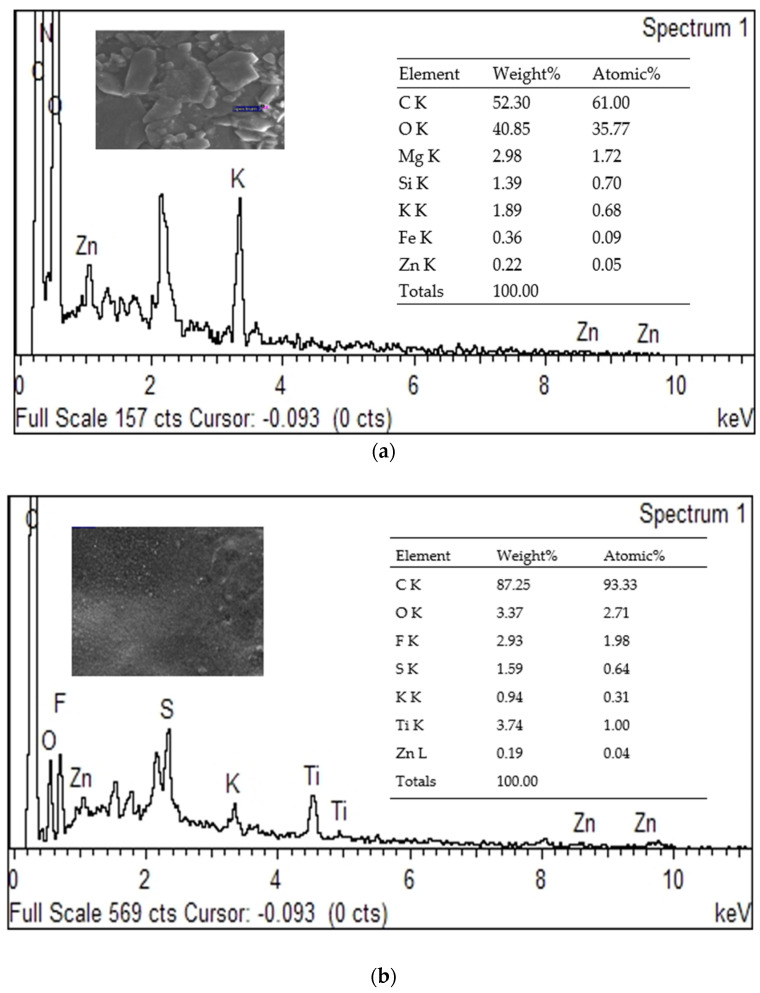
EDX of (**a**) concentrated latex (retentate); (**b**) PVDF-PVP-TiO_2_ membrane after filtration process.

The EDX results are presented in Figure 6a and display the constituent elements of concentrated latex after the filtration process, namely carbon (C), oxygen (O), potassium (K), zinc (Zn), magnesium (Mg), silica (Si), and iron (Fe). Carbon has the largest percentage, 52.30% wt, followed by oxygen (40.85% wt), while the element with the lowest percentage is zinc, with 0.22% wt. According to Danwanichakul et al. [5], raw rubber is made up of about 90–95% pure rubber, 1–2% fatty acids, 2–3% protein, 0.2–0.5% sugar, and 0.5% salts of sodium, potassium, magnesium, phosphorus, calcium, copper, manganese, and iron.

Figure 6b presents the elemental composition of a PVDF-TiO_2_-PVP membrane with a 16% wt PVDF concentration that is analyzed using EDX, following the filtration of skim latex, and reveals the presence of oxygen (O), carbon (C), potassium (K), titanium (Ti), fluorine (F), sulphur (S), and zinc (Zn). The presence of certain components suggests that the skim latex compounds have been deposited on the surface of the membrane, potentially leading to fouling. Carbon, as the predominant element (87% wt), is frequently encountered in organic substances, and its detection is likely attributed to organic constituents in the skim latex and the PVDF-PVP matrix. Oxygen (3.37% wt) is an essential chemical element in many senses, such as organic polymers and water. It is expected to be present due to the skim latex composition and the membrane material. The detection of potassium (K) in the EDX study may be attributed to the use of potassium-based chemicals or compounds during latex processing. The detection of titanium is linked to the existence of titanium dioxide nanoparticles within the PVDF-TiO_2_-PVP membrane. TiO_2_ is a frequently employed additive to augment the qualities of membranes, and it has the potential to be incorporated into the composition of these membranes. Zinc (Zn) is the least detected component (0.19% wt) and can be ascribed to using zinc-based compounds or additives during latex processing. Utilizing a PVDF-PVP-TiO_2_ membrane for filtering skim latex can lead to the capture of latex particles on the membrane’s surface. This phenomenon can reduce the ability of the membrane to allow substances to pass through and raise the differential in pressure on either side of the membrane.

## 4. Conclusions

This work provides a novel strategy for creating new starting membrane materials and using them as an alternate and effective method to treat natural rubber skim latex. The concentrated skim latex and skim serum have been obtained from the membrane ultrafiltration process. FESEM-EDX and FTIR analysis show the identification of skim latex deposits that contribute to fouling on the membrane surface. The presence of certain components suggests that the skim latex compounds have attached to the membrane surface, resulting in the accumulation of deposits. The CHNS analysis indicates that the membrane separation procedure effectively extracted a higher percentage of nitrogen, which represents the protein content, from skim latex compared to the skim serum obtained by the standard coagulation approach.

In addition, the skim serum obtained from the membrane filtration process did not identify the presence of sulphur, whereas the skim serum obtained from the acid coagulation process revealed a sulphur component of 0.367%. The use of a substantial quantity of sulfuric acid is responsible for the negative environmental impact, as it exacerbates the issue of wastewater and leads to a deterioration in the quality of the skim. Utilizing a PVDF-PVP-TiO_2_ membrane for the filtration of skim latex can result in the entrapment of latex particles on the surface of the membrane. Compared to the PVDF pristine membranes, the PVDF–PVP-TiO_2_ mixed-matrix membrane had a maximum skim serum flux of 12.72 L/m^2^h, while the PVDF pristine membrane had a lower flux of 8.14 L/m^2^h.

Further studies can focus on examining the effects of different operating pressures and temperatures and evaluating the membrane’s long-term stability, which would provide a deeper understanding of its practical performance and reliability. Further analysis of the fouling mechanism at different stages of filtration would also help in developing effective membrane cleaning strategies.

## Figures and Tables

**Table 1:** Skim latex properties.

Properties	Unit	Results
Total Solid Content	(%)	6.996
pH		9.590
Alkalinity	(%)	0.244

**Table 2:** Skim latex and concentrate skim latex properties.

Latex Type	TSC (wt %)	Alkalinity	pH	Brookfield Viscosity (cPs)
Skim Latex	6.996	0.244	9.590	50
Concentrate Skim Latex	7.67	0.546	9.34	133.33

## Data Availability

The original contributions presented in this study are included in the article. Further inquiries can be directed to the corresponding authors.

## Abbreviations

The following abbreviations are used in this manuscript:

TiO_2_	Titanium dioxide
PVDF	Polyvinilidene fluoride
PVP	Polyvinylpyrrolidone
NP	Nanoparticle
MMM	Mixed-matrix membrane
NIPS	Non-solvent phase inversion method
CHNS	Carbon, hydrogen, nitrogen, and sulphur
TSC	Total solid content
FTIR	Fourier transform infrared spectroscopy
FESEM	Field emission scanning electron microscopy
EDX	Energy-dispersive X-ray
